# Supplementary material for: Characterization of Bacillus Strains from Natural Honeybee Products with High Keratinolytic Activity and Antimicrobial Potential
Source: Microorganisms. 2023 Feb 11;11(2):456. doi: 10.3390/microorganisms11020456 (PMC9959047; doi:10.3390/microorganisms11020456)
Supplement: Supplementary file 1 [file microorganisms-11-00456-s001.zip › microorganisms-2213446-supplementary.pdf]

Table S1. **Antimicrobial Potential**. Gene clusters associated with the synthesis of nonribosomal peptide-synthetases (NRPS), polyketide synthases (PKSs) and ribosomally synthesized and post-translationally modified peptides (RiPPs) identified in the genomes of the *Bacillus* isolates AB6, CG1, X1 and Z1.

Strain	Source	Gene Cluster	Antibiotic#	Similarity (%)*	Reference
B. sonorensis AB6	Honey	NRPS	Lichenysin	100	(61)
		NRPS	Bacitracin	88	(62)
		NRPS	Bacillibactin, paenibactin, griseobactin	46	(63)
		RiPP, Lantipeptide	Cytolysin	40	(64)
		NRPS	Fengycin	30	(65)
		RiPP, Lantipeptide	Streptin	20	(66)
		NRPS-PKS	Plipastatin	23	(67)
		NRPS	Plipastatin- Fengycin	15	(65, 67)
		RiPP, Lassopeptide	ND	-	(68)
B. licheniformis CG1	Honey	NRPS	Lichenysin	100	(61)
		RiPP, Lantipeptide	Lichenicidin	100	(57)
		NRPS	Bacillibactin, griseobactin	46	(63)
B. sonorensis X1	Royal Jelly	NRPS	Lichenysin	100	(61)
		NRPS	Bacitracin	88	(62)
		NRPS	Bacillibactin, paenibactin, griseobactin	46	(63)
		NRPS	Fengycin	30	(65)
		NRPS-PKS	Plipastatin	23	(67)
		NRPS	Plipastatin- Fengycin	23	(65, 67)
		RiPP, Lantipeptide	Streptin	20	(66)
		RiPP, Lassopeptide	ND	-	(68)
B. subtilis Z1	Honey	NRPS	Fengycin	100	(65)
		RiPP, sactipeptide	Sporulation killing factor, skfA	100	(69)
		RiPP, sactipeptide	Subtilosin A	100	(70)
		NRPS-PKS	Bacillaene	92	(71)
		NRPS	Bacillibactin	92	(63)
		NRPS	Bacilysin	85	(72)
		NRPS	Surfactin	78	(73)
		RiPP, glyocin	Sublancin	66	(74)

#ND, not described; *Percentage of similar genes found in other clusters

57. Begley, M.; Cotter, P.D.; Hill, C.; Ross, R.P. Identification of a novel two-peptide lantibiotic, lichenicidin, following rational genome mining for LanM proteins. *Appl. Environ. Microbiol.* **2009**, *75*, 5451–5460.
61. Yakimov, M.M.; Timmis, K.N.; Wray, V.; Fredrickson, H.L. Characterization of a new lipopeptide surfactant produced by thermotolerant and halotolerant subsurface Bacillus licheniformis BAS50. *Appl. Environ. Microbiol.* **1995**, *61*, 1706–1713.
62. Eppelmann, K.; Doekel, S.; Marahiel, M.A. Engineered biosynthesis of the peptide antibiotic bacitracin in the surrogate host Bacillus subtilis. *J. Biol. Chem.* **2001**, *276*, 34824–34831.
63. May, J.J.; Wendrich, T.M.; Marahiel, M.A. The dhb operon of Bacillus subtilis encodes the biosynthetic template for the catecholic siderophore 2,3-dihydroxybenzoate-glycine-threonine trimeric ester bacillibactin. *J. Biol. Chem.* **2001**, *276*, 7209–7217.
64. Coburn, P.S.; Gilmore, M.S. The Enterococcus faecalis cytolysin: a novel toxin active against eukaryotic and prokaryotic cells. *Cell Microbiol.* **2003**, *5*, 661–669.
65. Koumoutsi, A.; Chen, X.H.; Henne, A.; Liesegang, H.; Hitzeroth, G.; Franke, P.; Vater, J.; Borriss, R. Structural and functional characterization of gene clusters directing nonribosomal synthesis of bioactive cyclic lipopeptides in Bacillus amyloliquefaciens strain FZB42. *J. Bacteriol.* **2004**, *186*, 1084–1096.
66. Wescombe, P.A.; Tagg, J.R. Purification and characterization of streptin, a type A1 lantibiotic produced by Streptococcus pyogenes. *Appl. Environ. Microbiol.* **2003**, *69*, 2737–2747.
67. Umezawa, H.; Aoyagi, T.; Nishikiori, T.; Okuyama, A.; Yamagishi, Y.; Hamada, M.; Takeuchi, T. Plipastatins: new inhibitors of phospholipase A2, produced by Bacillus cereus BMG302-fF67. I. Taxonomy, production, isolation and preliminary characterization. *J. Antibiot. (Tokyo)* **1986**, *39*, 737–744.
68. Hegemann, J.D.; Zimmermann, M.; Xie, X.; Marahiel, M.A. Lasso peptides: an intriguing class of bacterial natural products. *Acc. Chem. Res.* **2015**, *48*, 1909–1919.
69. Allenby, N.E.; Watts, C.A.; Homuth, G.; Pragai, Z.; Wipat, A.; Ward, A.C.; Harwood, C.R. Phosphate starvation induces the sporulation killing factor of Bacillus subtilis. *J. Bacteriol.* **2006**, *188*, 5299–5303.
70. Babasaki, K.; Takao, T.; Shimonishi, Y.; Kurahashi, K. 1985. Subtilosin A, a new antibiotic peptide produced by Bacillus subtilis 168: isolation, structural analysis, and biogenesis. *J. Biochem.* **1985**, *98*, 585–603.
71. Patel, P.S.; Huang, S.; Fisher, S.; Pirnik, D.; Aklonis, C.; Dean, L.; Meyers, E.; Fernandes, P.; Mayerl, F. Bacillaene, a novel inhibitor of procaryotic protein synthesis produced by Bacillus subtilis: production, taxonomy, isolation, physico-chemical characterization and biological activity. *J. Antibiot. (Tokyo)* **1995**, *48*, 997–1003.
72. Rogers, H.J.; Newton, G.G.; Abraham, E.P. Production and purification of bacilysin. *Biochem. J.* **1965**, *97*, 573–578.
73. Arima, K.; Kakinuma, A.; Tamura, G. Surfactin, a crystalline peptidelipid surfactant produced by Bacillus subtilis: isolation, characterization and its inhibition of fibrin clot formation. *Biochem. Biophys. Res. Commun.* **1968**, *31*, 488–494.
74. Paik, S.H.; Chakicherla, A.; Hansen, J.N. Identification and characterization of the structural and transporter genes for, and the chemical and biological properties of, sublancin 168, a novel lantibiotic produced by Bacillus subtilis 168. *J. Biol. Chem.* **1998**, *273*, 23134–23142.
